# Economic impact of generic antiretrovirals in France for HIV patients’ care: a simulation between 2019 and 2023

**DOI:** 10.1186/s12913-022-07859-w

**Published:** 2022-04-27

**Authors:** Romain Demeulemeester, Nicolas Savy, Michaël Mounié, Laurent Molinier, Cyrille Delpierre, Pierre Dellamonica, Clotilde Allavena, Pascal Pugliesse, Lise Cuzin, Philippe Saint-Pierre, Nadège Costa

**Affiliations:** 1 University of Toulouse III, 31330 Toulouse, France; 2 Health Economics Unit, Medical Information Department, University Hospital of Toulouse, Toulouse, France; 3 UMR 1295, National Institute for Health and Medical Research, Toulouse, France; 4 Faculté de Médecine, Université Paul Sabatier, INSERM, UMR 1295, 37 allées Jules Guesde, 31000 Toulouse, France; 5 CNRS UMR 5219, Toulouse Mathematics Institute, Toulouse, France; 6 Infectious Diseases Department, University of Côte d’Azur, University Hospital of Nice, Nice, France; 7 Infectious Diseases Department, University Hospital of Nantes, Nantes, France; 8 Infectious and Tropical Diseases Department, University Hospital of Martinique, Fort-de-France, France

**Keywords:** HIV, Generic antiretrovirals, Economic evaluation, Simulations, Agent-based model

## Abstract

**Background:**

In a context where the economic burden of HIV is increasing as HIV patients now have a close to normal lifespan, the availability of generic antiretrovirals commonly prescribed in 2017 and the imminence of patent expiration are expected to provide substantial savings in the coming years. This article aims to assess the economic impact of these generic antiretrovirals in France and specifically over a five-year period.

**Methods:**

An agent-based model was developed to simulate patient trajectories and treatment use over a five-year period. By comparing the results of costs for trajectories simulated under different predefined scenarios, a budget impact model can be created and sensitivity analyses performed on several parameters of importance.

**Results:**

The potential economic savings from 2019 to 2023 generated by generic antiretrovirals range from €309 million when the penetration rate of generics is set at 10% to €1.5 billion at 70%. These savings range from €984 million to €993 million as the delay between patent and generic marketing authorisation varies from 10 to 15 years, and from €965 million to €993 million as the Negotiated Price per Unit (NPU) of generics at market-entry varies from 40 to 50% of the NPU for patents.

**Discussion:**

This economic savings simulation could help decision makers to anticipate resource allocations for further innovation in antiretrovirals therapies as well as prevention, especially by funding the Pre-Exposure Prophylaxis (PrEP) or HIV screening.

**Supplementary Information:**

The online version contains supplementary material available at 10.1186/s12913-022-07859-w.

## Background

According to the World Health Organization, 36.5 million people worldwide were living with HIV in 2017 [1]. In France, people living with HIV (PHIV) were estimated to be 172,700 in 2016 [2]. Among them, 76% were receiving antiretroviral (ARV) treatments. The most recent figures from compulsory health insurances estimate the direct cost of HIV at €1.3 billion in 2017 [3]. ARV treatments accounted for 71% of these costs. Over the past 20 years, HIV has become a chronic disease due to successful HIV therapeutics [4]. The economic burden of HIV care will increase over the coming years because PHIVs on active antiretroviral regimens are now expected to have a close to normal lifespan [5, 6].

The European Medicines Agency (EMA) defines a generic drug as one that contains the same active substance(s) as the reference medicine and is used at the same dose(s) to treat the same disease(s). However, the drug name, the appearance (such as colour or shape) and packaging can be different from those of the reference medicine [7]. In France, a GD is defined as “a copy of an original medicinal drug whereby production and marketing are made possible by the expiry of the patent covering the innovator product” [8]. Additionally, the French Public Health Code defines a GD as “a specialty which is essentially similar and presents the same qualitative and quantitative composition of active ingredients, the same dosage form and bioequivalence as the original product” [8].

The last Association for Accessible Medicines (AAM) report shows that $265 billion were saved in 2017 for all payers thanks to GDs, and the 10-year savings were estimated at $1.19 trillion [9]. A 2014 report by the Intercontinental Marketing Services Institute for Healthcare Informatics shows that GDs provided savings of €100 billion a year for health systems in Europe [10]. Reports from the French Economic Committee for Health Products (CEPS) based on retrospective national databases of drug reimbursement statistics show that between 2010 and 2014, the use of GDs allowed the French health system to save €7 billion [11].

In the coming years, ARV generics launched on the market in 2017 (e.g. [ABC + 3TC]) as well as other molecules coming off patents (e.g., TDF) will provide an opportunity to improve efficiency in the context of resource allocation constraints. Two European studies estimated the five-year economic savings resulting from ARV generics at €187 million in Italy and €1.25 billion in the UK [12, 13]. These studies used population-based methods.

As an alternative to the usual population-based strategies, the agent-based approach aims to pinpoint the natural behaviour of a system from data [14, 15]. This behaviour can then be simulated to gain insight into the future of the system in a low-cost and time-saving manner. The main simulation lines are split into two phases: First, a group of patients (real or virtual patients [16]) is considered. Second, the behaviour of each patient’s outcomes over time and under predefined scenarios is simulated using predictive models commonly referred to as execution models [17]. There is a wide range of applications for agent-based models (ABM) [18], especially in Biology [19] and Economics [20], while they are fewer in the field of medical research [21, 22].

In France, robust real-life data are available to implement ABM to simulate potential savings from the imminent arrival of generic ARVs on the French market [23]. Our hypothesis is that these soon-to-be available generic ARVs will generate significant cost savings for the French National Health Insurance (FNHI), which entirely supports the care costs of PHIVs in the context of the Long-term Diseases scheme. The aim of this study is to estimate the economic impact on the FNHI, of the entry of generic ARVs on the French market from 2019 to 2023, according to different scenarios.

## Material and methods

### Data sources

Several data sources were used to implement the budget impact model using the ABM and are described below.

Nadis is an electronic medical record currently implemented in 22 French hospital centres and was developed for two purposes [23]. First, it allows the real-time collection of therapeutic information and results from biological and medical examinations that can then be shared between the different care units involved in the follow-up of PHIVs. After providing written consent, all PHIVs followed in any of the Nadis-using centres are enrolled. Second, the collected information provides a database that is representative of the French population of PHIVs under treatment and can be used for epidemiological, clinical, and therapeutic studies. To this end, the Dat’Aids group freezes the Nadis database annually and gathers data that is required for research projects. The specific extraction provided by the Dat’Aids group for this study was frozen in the Nadis database on December 31st, 2015. Hereafter, this will be referred to as the Dat’Aids database. For each patient, we accessed the covariates listed in Table 1 from the Dat’Aids database.

**Table 1:** State variables of the agents

Age	Years
Duration of the current treatment	Years
Duration of the HIV infection	Years
Country of birth	France
	Other
Gender	Male
	Female
Transmission mode	MSM
	Other
Cardiovascular disease	Yes
	No
Diabetes	Yes
	No
AIDS diagnosis	Yes
	No
Creatinine clearance (mL / min / 1.73 m^2^)	Low (≤ 29)
	Medium (> 29 and ≤ 89)
	High (> 89)
HIV RNA (viral load, in copies / mL)	Low (≤ 50)
	Medium (> 50 and ≤ 10,000)
	High (> 10,000)

*MSM* indicates Man who has Sex with Man, *AIDS* Acquired Immunodeficiency Syndrome, *HIV* Human Immunodeficiency Virus, *RNA* RiboNucleic Acid

The Medic’AM databases are national databases published yearly on the statistics for all medicines reimbursed by the FNHI [24]. To be more specific, they contain the total reimbursement base, the total amount reimbursed, and count of each medicine, by year and CIP 13 code. This database is an aggregate, which means that it contains no patient data. In this study, the counts of reimbursed medicines were used to estimate the baseline penetration rate of generic ARVs for 2019.

In France, the Marketing Authorisation delivered by the National Agency for Medicines and Health Products Safety (ANSM) is required for commercialisation of a drug. In order to implement the ABM, the Marketing Authorisation Dates (MAD) of patents and generic ARVs that are already available were collected from the ANSM compendium of proprietary drugs [25].

In order to assess the FNHI cost of ARVs, the Negotiated Prices per Unit (NPU) of patented ARVs and generic ARVs already available on January 1st, 2019 were collected from the Medicine and FNHI Tariff Information database (BdM-IT) [26].

### Population

The Dat’Aids database contained information on 31,722 patients. Among them, 27,341 had a complete record of every covariate included in the model for both the first and second semesters of 2015. Therefore, only the information on these patients was kept to train the ABM. All matched the following criteria:

- Being included in the Dat’Aids database
- Living with HIV
- Being under an ARV treatment
- 18 years old or more

In 2016, it was estimated that 131,252 French individuals lived with HIV and were on ARV treatment [2]. In addition, the number of patients who discover that they have the HIV infection was estimated to be approximately 6000 per year in France [2]. Since the standard percentage of newly infected patients who initiate an ARV treatment is approximately 76%, this results in a French population of around 140,000 HIV infected patients on ARV treatment in 2019. Therefore, the 27,341 patients included in our cohort represent around 19% of that population.

### The budget impact model (BIM)

Budget impact analyses estimate the financial consequences of the adoption and diffusion of a new strategy or technology by the healthcare system [27–29]. By using this model, the potential additional cost or cost savings of generalising a medical strategy can be quantified. This can in turn be used to address questions of affordability by measuring the financial incidence on a specific payer’s budget for implementing or removing the strategy, the rate of diffusion, possible substitution, and the size of the population concerned by the disease.

The economic impact of soon-to-be generic ARVs on the French market will be evaluated in relation to the FNHI. Only the cost of ARVs will be considered. To do so, the BIM developed in this study is an agent-based model, which was trained to pinpoint the behaviour of individual characteristics of PHIV from pre-existing data sources. Once trained, the ABM was then used to simulate the change in each PHIVs’ characteristics over the study period from 2019 to 2023 by stages of 6 months. At least one measurement of each PHIV’s viral load and CD4 cell count should be entered in the Nadis database for each six-month period [24]. Therefore, each patient’s data is expected to be updated at least once per semester, which motivated the choice of stages to ensure the availability of sufficient information to train the ABM. For each patient, the model provided outcomes for a set of state variables, including treatment regimen computed at each stage. It should also be noted that transitions toward an absence of treatment were also considered and determined from the Dat’Aids database so that possible non-adherence of PHIVs to their treatment could be integrated. A list of every state variable included in the ABM is provided in Table 1.

The execution models used to simulate the changes in covariates can be split into three categories:

- Time-fixed covariates (e.g., sex),
- Deterministic time-dependent covariates (e.g., age),
- Time-varying covariates.

**Fig. 1:**
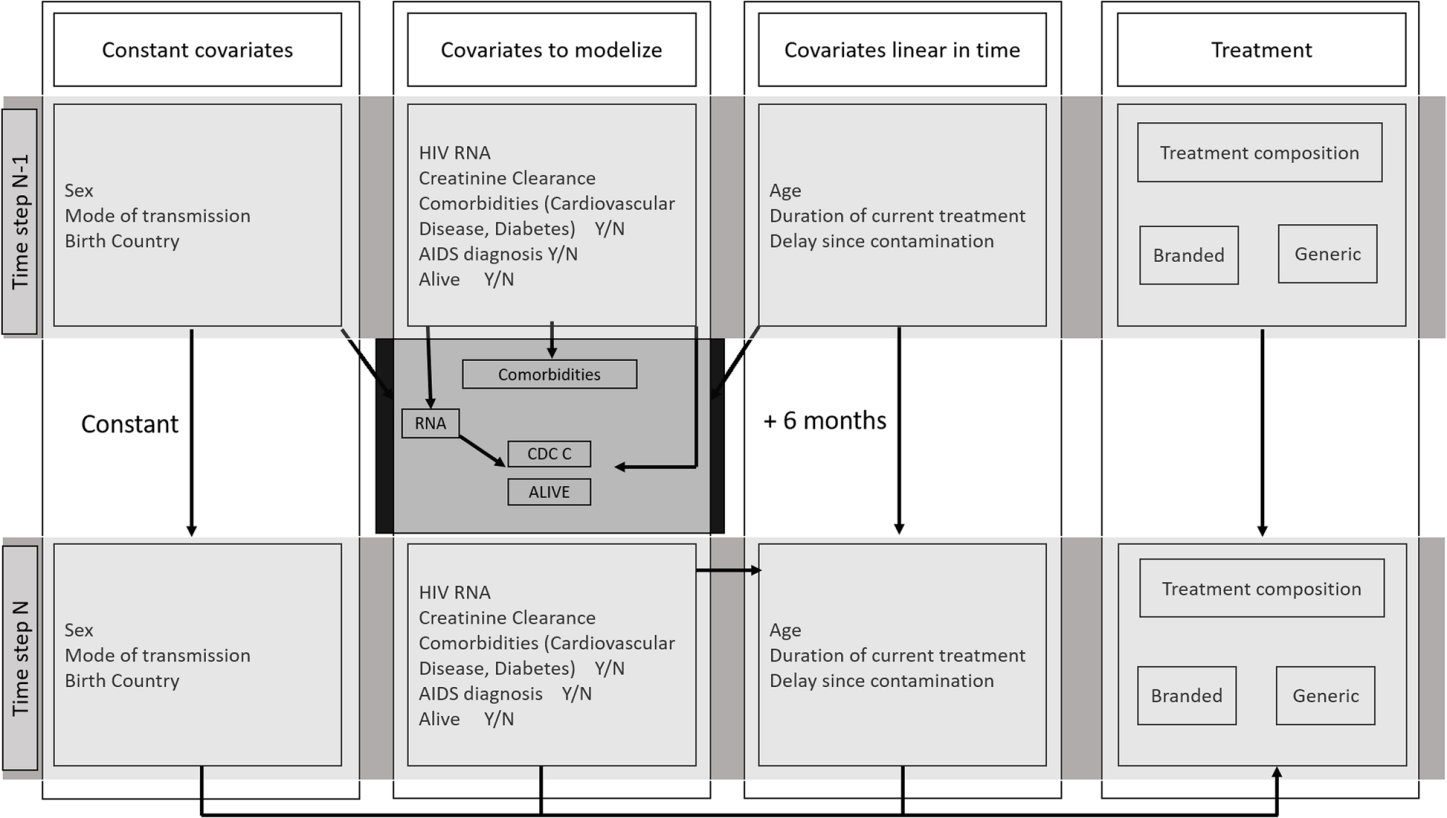
Updating scheme of covariates between step N-1 and N of the process. Scheme of the interaction between covariates during the transition from stage N-1 to stage N

Time varying covariates involve Markov chains with deterministic transitions (e.g., AIDS diagnosis) or random transitions provided by logistic regressions (e.g., HIV RNA). The scheme of the execution process in a single stage is provided in Fig. 1.

A more detailed description of the model is given in Online Resource 1, following the Overview, Design concepts and Details (ODD) protocol [30].

Once every treatment trajectory was obtained through the agent-based model, the cost savings from generic ARVs were evaluated by computing a differential cost over the five-year period that can be derived from a comparison between two scenarios. In the first scenario, only brand-name versions of ARVs were allowed as components of the treatment. In the second scenario, substitution of a brand-name by a generic ARV was allowed. The list of generic ARVs was updated at any stage to include those that were supposed to become available during the study period, depending on the MADs of patented ARVs.

### Investigated scenarios

Scenarios focused on the impact of several parameters of major importance on cost savings as defined below.

First, in France, the Marketing Authorisation delivered by the ANSM is required for commercialisation of a drug. However, to allow pharmaceutical firms to benefit from their investments in the development of new drugs and to ensure the protection of intellectual property, marketing authorisation cannot be granted for a generic drug before expiry of the patent for the brand-name version. The minimum period for such patents is 10 years as of the MAD and can be extended to 15 years by a Complementary Certificate of Protection [8].

Second, the penetration rate of a drug is defined as the ratio between the number of patients who take that specific drug and the total number of potential consumers. It is a relevant indicator of how much a drug might be used. The sales for each patented drug and of their generic versions over the year 2019 were extracted from the Medic’AM database [24]. The penetration rate for the generic version of each drug was then estimated by the ratio of the number of generic products and the number of both generic and associated patented drug products sold in 2019. The mean estimated penetration rates for all brand-name drugs with generic versions already available was 45%.

Third, in France, the NPU of a generic drug is usually set at 40% of its patent NPU at the time of entry on the market, while the NPU of the patent drug also decreases by 20%. In addition, based on the mean yearly rates observed from 2013 to 2018 [31], a 3.8% discount in drug NPU is applied for each year of follow-up.

### Analyses

For all the brand-name drugs for which a GD is not yet available, the parameters described above are unknown and consequently, assumptions must be made to obtain results. Therefore, we first parametrised a basic case scenario to build our BIM before conducting sensitivity analyses on each parameter.

For that basic case scenario, we set the time between the MAD of the patent and generic drugs, and the penetration rate of generics to respectively 13 years and 40%, which were estimated using available data [24]. The NPU of each generic drug was set at 40% of the NPU of its brand-name version.

Sensitivity analyses were conducted to examine the effect of each parameter on the BIM. A “One-At-a-Time” strategy was used, which consists of keeping all but one of the parameters at their reference value while varying that one parameter within a given range. The penetration rate was varied from 10 to 70% by increments of 15%, the MAD was varied from 10 to 15 years by increments of 1 year, and the NPUs of GDs were varied from 40 to 50% of the corresponding brand-name drug NPU by increments of 5%.

The results are given as the mean and standard deviation, along with a 95% bootstrap confidence interval derived from 100 simulation runs of the process. In addition, as our final population is assumed to represent approximately 19% of the total population of HIV-infected patients on ARV treatment, the total cost savings results was multiplied by 5.13 to rescale them according to the French population. Finally, all costs were expressed in 2019-euros.

## Results

### Sample characteristics

The baseline characteristics of our sample are presented in Table 2.

**Table 2:** Baseline characteristics of the Dat’Aids sample of patients

		*N* = 27,341		
		Mean	SD	95% bca CI
Age	Years	49.43	11.37	[49.29; 49.55]
Duration of the current treatment	Years	8	5.26	[7.94; 8.07]
Duration of the HIV infection	Years	30.96	17.09	[30.63; 31.06]
		N	%	
Country of birth	France	11,290	41	
	Other	16,051	59	
Gender	Male	18,880	69	
	Female	8461	31	
Transmission mode	MSM	10,610	39	
	Other	16,731	61	
Cardiovascular disease	Yes	8693	32	
	No	18,648	68	
Diabetes	Yes	1544	6	
	No	25,797	94	
AIDS diagnosis	Yes	6633	24	
	No	20,708	76	
Creatinine clearance (mL / min / 1.73 m^2^)	Low (≤ 29)	214	1	
	Medium (> 29 and ≤ 89)	14,457	53	
	High (> 89)	12,670	46	
HIV RNA (viral load, in copies / mL)	Low (≤ 50)	25,294	92	
	Medium (> 50 and ≤ 10,000)	1619	6	
	High (> 10,000)	428	2	

*SD* indicates Standard Deviation, *bca CI* bias-corrected and accelerated bootstrap Confidence Interval, *MSM* Man who has Sex with Man, *AIDS* Acquired Immunodeficiency Syndrome, *HIV* Human Immunodeficiency Virus, *RNA* RiboNucleic Acid

The mean age of patients was 49.93 years (SD: 11.37) and the majority were men (69%). The proportion of patients with a diagnosis of AIDS was 24, 92% of whom had a low viral load. Concerning comorbidities, only 6% of the patients had diabetes, but approximately half of these patients (54%) had renal failure, be it moderate or severe, and 32% had a cardiovascular disease.

### Basic case scenario

The results obtained from the basic case scenario are presented in Table 3 in the form of cumulative sums over the 10 semesters of the study period. The five-year cost savings amounted to €993.05 million (M€) [987.36; 1004.82].

**Table 3:** Cumulated total cost savings in five years for the French population in 2019-M€ (mean and standard deviation (SD)) together with its 95% bootstrap confidence interval (from 100 simulation runs) from the basic case scenario

Year	Mean (2019-M€)	SD	95% BCI
1	190.19	1.28	[188.33; 192.35]
2	382.43	1.99	[380.10; 386.02]
3	569.95	2.76	[566.49; 575.29]
4	785.23	3.58	[781.18; 793.12]
5	993.05	5.19	[987.36; 1004.82]

*M€* indicates millions of euros, *SD* Standard Deviation, *BCI* Bootstrap Confidence Interval

### Sensitivity analyses

The variations in total five-year cost savings and in the average cost savings per patient per year induced by the variations in the penetration rate, the MAD of generic drugs and their NPUs are shown in Table 4. When reading the results of sensitivity analyses, the reader should bear in mind that when one of the three parameters varies, the other two are held constant and set at the basic case value, i.e., 40% for the penetration rate, 13 years from the MAD of brand-name drugs for the MAD of generic drugs, and 40% of the NPU of brand-name drugs for the NPU of generic drugs.

**Table 4:** Variations in total five-year cost savings and average cost savings per patient per year, in respectively 2019-M€ and 2019-€ (mean and standard deviation (SD)) together with their 95% Monte Carlo confidence intervals, according to the penetration rate, marketing authorisation date of generics and NPUs of generics (from 100 simulation runs)

		Total differential cost in five years (2019-M€)			Average differential cost per patient per year (2019-€)		
		Mean	SD	95% CI	Mean	SD	95% CI
Penetration rate	10%	309.31	2.24	[305.72; 313.26]	410.52	616.83	[0; 2053.97]
	25%	684.14	3.56	[679.82; 691.79]	910.05	892.42	[0; 2936.21]
	40%	993.05	5.19	[987.36; 1004.82]	1323.39	1047.35	[0; 3343.42]
	55%	1264.29	5.44	[1259.35;1276.74]	1687.06	1164.69	[0; 3567.24]
	70%	1508.18	6.78	[1501.84; 1523.80]	2015.19	1276.51	[161.58; 3814.33]
Time between brand-name and generic MAD	+ 10	901.19	4.69	[902.39; 911.22]	1199.28	1041.79	[0; 3272.08]
	+ 11	896.10	4.59	[890.27; 906.06]	1191.66	1040.35	[0; 3268.44]
	+ 12	894.49	4.60	[888.69; 904.44]	1188.99	1039.65	[0; 3262.67]
	+ 13	993.05	5.19	[987.36; 1004.82]	1323.39	1047.35	[0; 3295.56]
	+ 14	944.86	4.82	[939.65; 955.64]	1259.49	1043.07	[0; 3285.88]
	+ 15	909.82	4.68	[904.12; 920.01]	1210.72	1043.03	[0; 3275.32]
NPU of generics	40%	993.05	5.19	[987.36; 1004.82]	1323.39	1047.35	[0; 3295.56]
	45%	979.11	5.09	[973.41; 990.63]	1304.35	1043.67	[0; 3287.96]
	50%	965.18	5.01	[959.46; 976.44]	1285.31	1040.84	[0; 3284.05]

*M€* indicates million euros, *SD* Standard Deviation, *CI* Confidence Interval, *MAD* Marketing Authorisation Date, *NPU* Negotiated Price per Unit

Both the total five-year cost savings and the average cost savings per patient per year increase with the penetration rate of generic drugs. They respectively range from M€309.31 and €410.52 with a penetration rate of 10% to M€1508.19 and €2015.19 with a penetration rate of 70%.

The total five-year cost savings (average cost savings per patient per year) first decreases from M€901.19 (respectively €1199.28) to M€894.49 (respectively €1188.99) as we parametrise generic MAD to 12 years after brand-name MAD instead of 10 years. When generic MAD is set at 13 years after brand-name MAD, these cost savings then increase to M€993.05 and €1323.39 before decreasing once again as the time between generic and brand-name MADs is set at 14 and 15 years.

The total and average cost savings decrease with the NPUs of generic drugs at their market entry being taken as higher percentages of brand-name drug NPUs. They range from M€993.05 (respectively €1323.39) to M€965.18 (resp. €1285.31). The total variation can be simply interpreted as a variation of 10% in the share of the total and average cost savings induced by using GDs that were not available before the study and on which we based hypotheses.

## Discussion

This budget impact model using an ABM, highlights the fact that the potential economic savings between 2019 and 2023 due to the introduction of generic ARVs are significant and mostly driven by the penetration rate of generics. They range from €309 million to €1.5 billion as penetration rates vary between 10 and 70%, from €894 million to €993 million as the time between patent and generic MAD varies between 10 and 15 years, and from €965 million to €993 million as generic NPUs vary between 40 and 50% of patent ARVs NPUs.

Only two studies were found in which models were used to estimate the economic impact of new generic antiretrovirals. Restelli et al. [12] developed a BIM to forecast the rates of use of brand and generic ARVs and their impact on the Italian National Health Service budget from 2015 to 2019. They estimated the five-year economic savings at €187 million. They used expert opinions to drive their model and to develop scenarios according to the introduction of generic drugs or new brand drugs. However, they did not consider the generic and brand drug costs and changes in penetration rates over time, the variability in generic drug marketing authorisation dates, or the patients’ individual characteristics and their ability to switch from one treatment to another several times. Contrary to this method, the one developed in our study puts individuals at the heart of predictions. Instead of learning, predicting, and applying ARV consumption rates to a population, regardless of individual specificities, it rather uses data from medical histories to learn and predict the changes in each individual’s characteristics and treatments. Therefore, this way of assigning treatments over time is closer to the constrained allocation of ARVs observed in real-life management of HIV. Hill et al. [13] estimated the economic impact of generics at €1.25 billion, using a comparison between two scenarios. In their basic case scenario, every patient was assigned to brand-name antiretrovirals while in the second scenario, they all switched to generic drugs. They used a UK Collaborative HIV Cohort database to estimate the proportions of patients taking each drug and the British National Formulary database to estimate drug costs. This study provides deterministic results without a sensitivity analysis. Nevertheless, this study was an oral presentation, and therefore, the limited amount of available information prevents us from making a more thorough comparison with our work.

The agent-based method proposed here in the context of health economics research has four major advantages. First, it makes it possible to integrate many more parameters in the prediction of cost savings, especially individual parameters together with their correlation structures, which makes the predictions more realistic. Second, the effect of time can be examined through longitudinal models. Third, the precision of predictions can be assessed by incorporating randomness into the dynamics of the system. This precision can be evaluated or illustrated by means of bootstrap confidence intervals derived from the distribution of the predictions obtained through several simulation runs. It is a precious tool in this context to compare and identify the main sources of randomness. Fourth, the individual behaviour of patients can be examined and therefore the conclusion can be modulated in terms of population together with individuals. The main drawback is usually modelling and comes from the choice of models and the performance of the calibration of such models. The choice of models is driven by the data and the clinical input on the disease. For this study, we benefited significantly from the help of the Dat’Aids scientific committee, comprised of experts in the management of HIV-infected patients.

This study has several limitations that must be discussed. The time required to clean the Dat’Aids database, design the ABM, and implement it was much more than we had imagined. Consequently, by the time we were able to produce the first results, we were near the end of the period during which we had initially planned to carry out simulations. Therefore, as the ABM was built for predictive purposes, we decided to change the simulation period to 2019–2023. We are aware that doing so with a model trained on 2015 data presents major drawbacks. First, we were unable to consider the ARVs that entered the French market after 2015 as we lacked the necessary data. This could lead to an overestimation of the cost savings as these new patent ARVs will not be genericised during the course of the study. Second, the baseline characteristics and treatments of the simulated patients were taken from the 2015 Dat’Aids data. Overcoming this limitation would entail retraining and running the model on a more recent extraction of the Nadis cohort, which, unfortunately, we were unable to do within a reasonable time frame. However, we were able to perform analyses on a 2019 Nadis extraction, and found that the distribution of the PHIVs’ characteristics did not differ from those of PHIVs included in the 2015 extraction. We also estimated that 18.9% of the PHIVs included in the 2019 Nadis extraction had treatment regimens containing an ARV that entered the French Market between 2016 and 2019. Approximately 83.5% were on an STR containing tenofovir alafenamide (TAF), and this was 15.8% of the PHIVs included in the 2019 Nadis extraction. The proportion of patients that switches from tenofovir disoproxil (TDF) to TAF can be expected to increase in the future as TAF has a lower toxicity than TDF, but we were unable to evaluate the share of patients that would have made that switch by 2023. Still, in light of these findings, using the 2015 PHIV data as a baseline for the simulations that start in 2019 is not likely to affect the model outcomes. In addition, we gathered the exact information on MADs, NPUs, and penetration rates for every generic ARV that entered the French market between 2015 and 2019. To stay as consistent as possible with the study period, the version of ARVs consumed by each PHIV in the cohort was randomly selected between patent and generic product according to the penetration rates of 2019.

We did not consider the changes in the value of the euro across the study period. In fact, drug NPUs in France are negotiated with the government. Therefore, it seemed improbable that they would be impacted by fluctuations in the euro. However, we accounted for changes in drug NPUs by applying a 3.8% yearly discount based on rates observed from 2013 to 2018.

This study only focused on ARV costs. It does not include all resources consumed by PHIVs such as other direct medical and non-medical costs, indirect or informal costs. The results presented here are based on the assumptions underlying the execution models which are detailed in the associated section in supplementary materials. Please note that we did not consider the ability of some patients to break their Single Tablet Regimen (STR) (i.e., switch from a one-pill combination of several medicines to several pills) as this would have resulted in a much higher complexity in the algorithm. Such a consideration ensures that when the generic version of a medicine that is also part of a combination is available, but the combination itself is not, patients are prevented from breaking it up to take the generic. Therefore, resulting cost savings may be underestimated. In addition, we conducted no analysis on the efficacy of generic drugs or their impact on health as we considered both the efficacy and the safety to be similar between brand-name and generic drugs. Walensky et al. [32] and Sweet et al. [33] used simulation models to compare both cost and efficacy between STRs based on a foundation of emtricitabine and tenofovir (FTC/TDF) and their multiple-tablet counterparts, including generics when available and exchanging lamivudine for emtricitabine (e.g., EFV/TDF/FTC vs. generic EFV + TDF + generic lamivudine). Both studies demonstrated a higher efficacy of brand-name STRs compared to generic-based multi-tablet regimens (gMTRs), mainly as a result of poorer adherence to gMTRs than to STRs because of the pill burden and a lower efficacy of lamivudine compared to emtricitabine. However, their cost evaluation differs significantly from ours as only a few brand-name STRs were studied, the focus was on the breaking of STR, and in both studies, the two scenarios that were compared were everyone taking a brand-name STR or everyone taking a gMTR.

## Conclusions

In France, the care costs of HIV-infected patients are entirely supported by the FNHI in the context of the Long-Term Diseases scheme. In a context of resource constraints, it is therefore of paramount importance to develop and encourage alternative therapeutic strategies to lower health expenses without lowering the quality of patient care. With an acknowledged similar effectiveness and lower cost compared to their brand-name counterparts, generic drugs meet both the above criteria. This study highlights significant potential savings due to the introduction of new generic ARVs in France, ranging from €309 million to €1.5 billion as the penetration rate of generics varies between 10 and 70%. As shown by the sensitivity analyses, the penetration rate is the most important parameter that drives economic savings. In fact, these savings range from €894 million to €993 million as the time between patent and generic MADs varies between 10 and 15 years, and from €965 million to €993 million as generic NPUs vary between 40 and 50% of patent ARV NPUs. French health care authorities must encourage healthcare professionals to prescribe generic ARVs as well as patient compliance with generics. Such results should be taken into consideration by policy makers and encourage them to keep promoting the use of GDs. As shown in the discussion section, those potential savings remain limited by the increasing number of STRs being developed by pharmaceutical firms. In fact, although this could provide more savings, breaking STRs to be able to benefit from generic ARVs could result in poorer adherence to treatment [34]. A possible option to address this issue would be to scale the NPUs of STRs containing ARVs with already available generics based on these GD NPUs. These estimates of savings may also help decision makers to anticipate future choices for the funding of secondary prevention, specifically to fund the Pre-Exposure Prophylaxis (PrEP) [35–37] or for the early screening of hidden HIV population such as migrants for example. They could also be used to provide headway for innovation, giving health care payers the possibility to reimburse innovative and expensive new medicines, especially in the field of infectious disease.

## Supplementary Information

**Additional file 1.**

## Data Availability

The Dat’AIDS database is constituted in real time by the Nadis® computerised medical record used by medical units which manages HIV-positive patients. All patients included in the Nadis® computerised medical record have signed a written consent, in accordance with the agreements of the National Data Protection Agency. Data are available upon authorisation of the scientific council of Dat’AIDS which can be solicited by consultation of one of its members or using the following e-mail address: contact@dataids.org. The project was presented to the Dat’AIDS scientific council on 20 November 2015, which authorised the use of the data. Anonymised data was transferred by secure messaging on March 8, 2016. Additional data, including updates, were transferred by secure messaging on 15 September 2016 and 2 December 2016.

## Abbreviations

3TC: Lamivudine
AAM: Association for Accessible Medicines
ABC: Abacavir
ABM: Agent-Based Model
ANSM: National Agency for Medicines and Health Products
ARV: Antiretroviral drug
BdM IT: Medicines and Tariff Information Database
BIM: Budget Impact Model
EFV: Efavirenz
EMA: European Medicines Agency
EMR: Electronic Medical Record
FNHI: French National Health Insurance
FTC: Emtricitabine
GD: Generic Drug
gMTR: Generic-Based Multiple Tablet Regimen
HBV: Hepatitis B Virus
HCV: Hepatitis C Virus
HIV: Human Immunodeficiency Virus
MAD: Marketing Authorisation Date
NPU: Negotiated Price per Unit
ODD: Overview Design and Details
PHIV: Patient living with HIV
PrEP: Pre-Exposure Prophylaxis
SD: Standard Deviation
STR: Single Tablet Regimen
TDF: Tenofovir Disoproxil Fumarate
