# Supplementary material for: Economic impact of generic antiretrovirals in France for HIV patients’ care: a simulation between 2019 and 2023
Source: BMC Health Serv Res. 2022 Apr 27;22:567. doi: 10.1186/s12913-022-07859-w (PMC9044646; doi:10.1186/s12913-022-07859-w)
Supplement: Supplementary file 1 — Additional file 1. [file 12913_2022_7859_MOESM1_ESM.pdf]

Title

The economic impact of generic antiretrovirals on HIV patient care in France: a simulation for 2019 to 2023

Running title

The economic impact of generic antiretrovirals

Journal

PharmacoEconomics

Authors

Romain Demeulemeester[1,2,3] : MSC

Nicolas Savy[1,4] : PhD

Michaël Mounié[2,3] : PhD

Laurent Molinier[1,2,3] : MD, PhD

Cyrille Delpierre[1,3] : PhD

Pierre Dellamonica[5] : MD, PhD

Clotilde Allavena[6] : MD

Pascal Pugliesse[5] : MD

Lise Cuzin[7,8] : MD

Philippe Saint-Pierre[1,4] : PhD

Nadège Costa[2,3] : PhD

1. University of Toulouse III, Toulouse 31330, France
2. Health Economic Unit, Medical Information Department, University Hospital of Toulouse, Toulouse, France
3. UMR 1027, National Institute for Health and Medical Research, Toulouse, France
4. CNRS UMR 5219, Toulouse Mathematics Institute, Toulouse, France
5. Infectious Diseases Department, University of Côte d'Azur, University Hospital of Nice, Nice, France
6. Infectious Diseases Department, University Hospital of Nantes, Nantes, France
7. Infectious and Tropical Diseases Department, University Hospital of Martinique, Fort-de-France, France
8. UMR 1295, National Institute for Health and Medical Research, Toulouse, France

Online Resource 1. Description of the Agent Based Model according to the Overview, Design Concepts and Details protocol

The Overview, Design concepts and Details (ODD) protocol published by Grimm et al. [31] in 2006 aims to standardise the description of the agent-based model (ABM) to make it more understandable to most readers. In this document, we follow the ODD protocol to describe an ABM we developed to simulate the behaviour of HIV infected patients (PHIV) from 2019 to 2023, and evaluate the economic impact of the introduction of generic antiretrovirals in France.

1. Purpose

The aim of the ABM described here is to simulate the follow up of PHIVs, and more precisely, the sequence of their treatment allocation. Each such treatment is defined as an ARV combination and whether each of the ARVs is taken as brand-name or a generic drug. Such outputs are further used to evaluate the follow-up cost of each patient in terms of treatment and to compare it to another follow up scenario in which only brand-name ARVs are prescribed, which allows an estimation of economic savings imputable to the entry of new generic ARVs on the French market.

2. Entities, state variables and scales

This model is only comprised of one type of agent, PHIVs. Their state variables are listed in table 1 included in the main manuscript. We should also consider the addition of the treatment as such a state variable. In fact, treatments are not directly adapted by PHIVs themselves, but rather by clinicians specialised in HIV management. However, considering that in France, all PHIVs are granted the same access to care in the context of the Long-Term Diseases scheme, it is better to consider treatment here as an adaptive trait rather than the result of an interaction with another type of entity (a clinician for example).

3. Process overview and scheduling

The scheduling of the process is presented in figure 2 in the form of an algorithm. The covariates and treatment updating phases of this algorithm are described in figure 1, included in the main manuscript. The process progresses by stages of six months over a five-year period. The state variables of each agent are re-evaluated at

each of these stages. The follow-up costs mentioned in the "Purpose" section are directly evaluated as the process advances.

Fig.2 Scheduling of the process

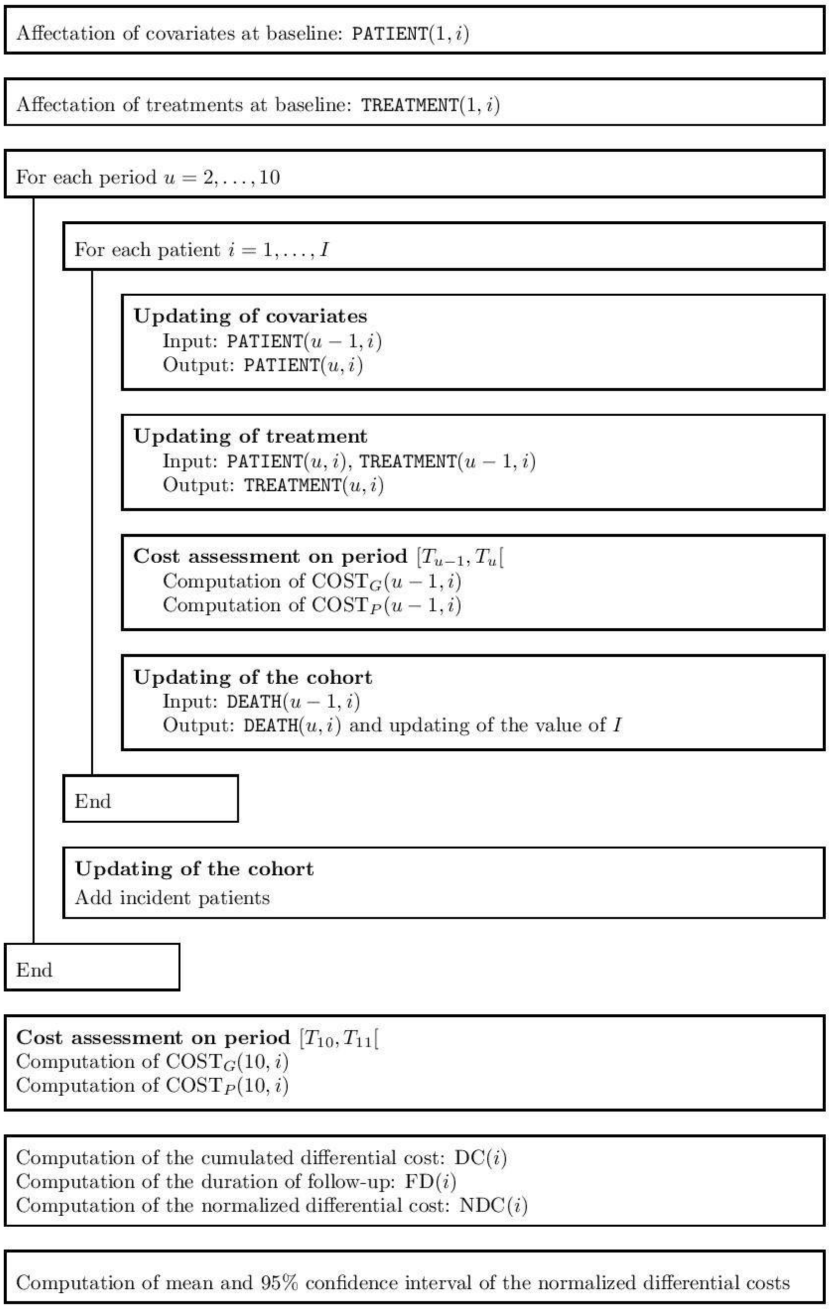

4. Design concepts

4.1. Emergence

The main phenomena that we expect to emerge from the model presented here is the adaptation of treatment composition during follow-up of PHIVs as well as the integration in these treatments of generic ARVs just entering the French market. In fact, the consumption of generic ARVs by the PHIV population cannot be easily anticipated even with a set penetration rate, because the ARV combinations of treatments change in a complex way.

4.2. Adaptation and fitness

As described above, the only adaptive trait that PHIVs have is their treatment. They do not seek to directly improve any measure of fitness. Instead, the model that adapts their treatment was trained to reproduce the allocations observed in the Dat'Aids database. It is assumed that such allocations are aimed at improving the patients' condition as they were chosen by HIV management experts.

4.3. Prediction

As a consequence of the changes in their treatment, the condition of PHIVs may also change over time. These changes are also modelled from data and will be discussed in further detail in later sections.

4.4. Sensing

Each PHIV is assumed to be aware of all his state variables at current and previous stages. Consequently, every state variable is taken into consideration during the updating of the treatment phase of the process.

4.5. Interaction

There is no interaction between PHIVs.

4.6. Observation

The data collected from the AMB are the treatment sequence of each PHIV, which include ARV combinations and whether branded or generic version of these ARVs is used.

5. *Initialisation*

The initial state of the cohort evolving through the ABM consists of the values of state variables identified in the Dat'Aids database on 31 December 2015, which includes 27,341 PHIVs. This setup is always the same at the start of every simulation run. However, incident cases are added at each stage of the process and are initialised by random drawing among the initial PHIVs of the cohort.

6. *Input data*

As described in the main manuscript, the model uses three hyperparameters as input. The first is the time between the marketing authorisation dates (MAD) of a brand-name drug and its generics. The second is the maximum penetration rates of generics. The actual penetration rate of a generic drug is set to grow linearly to the maximum penetration rate over the year following its entry on the French market. The last input is the percentage of brand-name drug rate that is used as the rate for generics on entry on the market. Sensitivity analyses on the cost outputs of the model can be conducted according to these inputs.

7. Sub-models

To describe the sub-models involved in the process, some notations (?) must be introduced. For the rest of this section, the correspondence between state variables and their abbreviations is indicated in table 5 below.

Table 5. Abbreviations of state variables.

State variables	Abbreviation
Age	AGE
Duration of the current treatment	$TREATD$
Duration of the HIV infection	$HIVD$
Country of birth	BC
Gender	SEX
Route of contamination	$CONTA$
Cardiovascular disease	$HEART$
Diabetes	$DIAB$
CDC stage C	$HIVS$
Creatinine clearance (mL / min / 1.73	$CREA$
HIV RNA (viral load, in copies / mL)	RNA
Patient being alive	$ALIVE$

For any given PHIV indicated by i, any state variable: V and for $u = 2, \dots, 10$, we will indicate by $V(u, i)$ the value of state variable V for PHIV i, at stage u.

7.1. *Step 1: Updating patients' covariates*

To update the patients' covariates at each stage, different models are involved depending on the nature of the covariates and the desired precision for the simulations:

Time-fixed covariates. For $u = 2, \dots, 10,$ we have $GENDER(u,.) = GENDER(1,.), BC(u,.) = BC(1,.)$ and $CONTA(u,.) = CONTA(1,.)$.

Deterministic time-dependent covariates. AGE and $HIVD$ change during patient follow-up because time is passes and these covariates are only a duration. The changes are only an increment of six months in the covariates. For $u = 2, \dots, 10,$ we have $AGE(u,.) = AGE(u-1,.) + 6$ and $HIVD(u,.) = HIVD(u-1,.) + 6$. $TREATD$ changes in the same way but must be reset to 0 in case the treatment is changed.

$$TREATD(u,.) = \begin{cases} TREATD(u-1,.) + 6 & \text{if there is no treatment switch,} \\ 0 & \text{if there is a change in treatment at time } T_u. \end{cases}$$

Time-varying covariates. Covariates $HIVS, HEART, DIAB$ and $ALIVE$ may change during patient follow-up. These changes can lead to a modification in patient treatment. The changes in these covariates is directed by Markov chains where the matrices of transition, indicated by $\boldsymbol{M}_{HEART}$, $\boldsymbol{M}_{DIAB}$, $\boldsymbol{M}_{HIVS}$ and $\boldsymbol{M}_{ALIVE}$ are chosen to be constant.

Time-varying covariates and randomness according to covariates. For RNA and $CREA$, the matrices of transition, indicated by $\boldsymbol{M}_{RNA}$ and $\boldsymbol{M}_{CREA}$ cannot be assumed to be constant because these transitions depend on patients' covariates. For these models, the probabilities of transition are modelled by logistic or polytomic regression. The covariates involved in the model are selected by a backward stepwise strategy.

For $RNA(u,.)$, the covariates involved are $RNA(u-1,.), GENDER(u-1,.)$, $AGE(u-1,.)$, $CONTA(u-1,.)$, $HIVS(u-1,.)$, $HIVD(u-1,.)$, $HEART(u-1,.)$, $IR(u-1,.)$ and $TREATD(u-1,.)$.

For $CREA(u,.)$, the covariates involved are $CREA(u-1,.)$, $GENDER(u-1,.)$, $AGE(u-1,.)$, $RNA(u-1,.)$, $HIVS(u-1,.)$, $HIVD(u-1,.)$, $HEART(u-1,.)$ and $TREATD(u-1,.)$.

Calibration of the execution models. Calibration of the models which means the estimation of the coefficients of $\boldsymbol{M}_{HEART}$, $\boldsymbol{M}_{DIAB}$, $\boldsymbol{M}_{HIVS}$ and $\boldsymbol{M}_{ALIVE}$ as well as the estimation of the parameters of the logistic (polytomic) regressions involved in the coefficients of $\boldsymbol{M}_{RNA}$ and $\boldsymbol{M}_{CREA}$ are derived from the DAT'AIDS database.

7.2. Step 2: Updating the treatment

Updating the treatment at time u, follows four rules:

- **Rule 1**: a patient can keep his treatment,
- **Rule 2**: a patient can switch from one treatment to another,
- **Rule 3**: a patient can switch to the generic version of his treatment,
- **Rule 4**: a patient who changes to a generic drug cannot change back to the brand-name drug as long as the treatment does not change.

The execution models, accounting for these rules, are defined as:

- Patients can switch for a treatment to another according to a Markov chain where the transition matrix is estimated from the DAT'AIDS database. For those transitions observed for a large number of patients in the DAT'AIDS, a logistic regression model is adjusted to model the transition probabilities as a function of some covariates. In this setting, the covariates involved are chosen case-by-case following a backward step-by-step strategy. When transitions are rare (less than 100 observations), the probability is considered as constant.

- Patients can switch to the generic version of their treatment. This happens with probability depending on time t represented by Figure 3 and defined by:

$$PROBCONV(t) = \begin{cases} 0 & \text{if } t < GMAD, \\ PENRATE \dfrac{t - GMAD}{PENTIME - GMAD} & \text{if } GMAD \leq t < PENTIME, \\ PENRATE & \text{if } t > PENTIME. \end{cases}$$

Where $PROBCONV$ denotes the probability at time t of a PHIV switching from the brand-name to the generic version of one of the ARVs in their treatment; $PENRATE$ indicates the maximum penetration rate, $GMAD$ the MAD of the generic drug and $PENTIME$ the time at which $PENRATE$ is reached. For the simulations of the main manuscript, $PENTIME$ was set to one year after the MAD of generics.

7.3. Step 3: Updating the cohort

At a given stage Tu, the cohort is updated by including 433 incident cases. This quantity was calculated with the literacy data given in the “Population” section. These initial state variable values of incident cases are randomly drawn from the initial DAT’AIDS cohort.

A patient i who died during the period $[u-1, u[$, which means $ALIVE(u,i) = 1$ and $ALIVE(u,i) = 0$, is not removed but all his future costs are set to 0.

Finally, let us denote $FD(i)$ the duration of the follow-up (in semester) for patient i.

7.4. Step 4: Computation of the differential cost

The differential cost DC for patient i is defined by:

$DC(i) \; = \; COST_B(i) - COST_G(i) = \sum_{u=1}^{10}(COST_B(u,i) - COST_G(u,i)),$

where $COST_B(i)$ represents the cost for patient i with no switch to generics and $COST_G(i)$ represent the cost assuming a pre-specified scenario of switching to generics. The main indicator of interest is the total differential cost in five years for the French population, defined as the sum divided by the patients for the individual differential cost $DC(i)$ times 19% which approximates the fraction of the French HIV population integrated in DAT'AIDS. Note that if a patient i died during the period $[u, u+1[$ his future costs are set to 0, which means that:

$COST_B(v,i) = COST_G(v,i) = 0$ for any $v \geq u+1$.

As described later, patients may die before the end of the follow-up and incident cases are possible. The differential cost should be normalised according to the follow-up duration to define the normalised differential cost:

$$NDC(i) = \frac{DC(i)}{FD(i)}.$$

Another indicator of interest is the average differential cost per patient per semester, defined as the average divided by the patients for the values of $NDC(i)$.
